# Analysis and classification of oncology activities on the way to workflow based single source documentation in clinical information systems

**DOI:** 10.1186/s12911-015-0231-x

**Published:** 2015-12-22

**Authors:** Stefan Wagner, Matthias W. Beckmann, Bernd Wullich, Christof Seggewies, Markus Ries, Thomas Bürkle, Hans-Ulrich Prokosch

**Affiliations:** Chair of Medical Informatics at the Friedrich-Alexander-University Erlangen-Nuremberg, Am Wetterkreuz 13, D-91058 Erlangen-Tennenlohe, Germany; Department of Anaesthesiology, University Hospital Erlangen, Krankenhausstraße 12, D-91054 Erlangen, Germany; Comprehensive Cancer Center Erlangen-EMN, University Hospital Erlangen, Östliche Stadtmauerstraße 30, D-91054 Erlangen, Germany; Department of Obstetrics and Gynecology, University Hospital Erlangen, Universitätsstraße 21-23, D-91054 Erlangen, Germany; Department of Urology, University Hospital Erlangen, Maximiliansplatz 2, D-91054 Erlangen, Germany; Medical Informatics and Communication Center, University Hospital Erlangen, Glückstraße 11, D-91054 Erlangen, Germany; Department for Organizational Development, Klinikum Nuremberg, Prof.-Ernst-Nathan-Str. 1, D-90419 Nuremberg, Germany; Institute for Medical Informatics I4MI, Bern University of Applied Sciences BFH, Höheweg 80, CH-2502 Biel/Bienne/Bern, Switzerland

## Abstract

**Background:**

Today, cancer documentation is still a tedious task involving many different information systems even within a single institution and it is rarely supported by appropriate documentation workflows.

**Methods:**

In a comprehensive 14 step analysis we compiled diagnostic and therapeutic pathways for 13 cancer entities using a mixed approach of document analysis, workflow analysis, expert interviews, workflow modelling and feedback loops. These pathways were stepwise classified and categorized to create a final set of grouped pathways and workflows including electronic documentation forms.

**Results:**

A total of 73 workflows for the 13 entities based on 82 paper documentation forms additionally to computer based documentation systems were compiled in a 724 page document comprising 130 figures, 94 tables and 23 tumour classifications as well as 12 follow-up tables. Stepwise classification made it possible to derive grouped diagnostic and therapeutic pathways for the three major classes

- solid entities with surgical therapy

- solid entities with surgical and additional therapeutic activities and

- non-solid entities.

For these classes it was possible to deduct common documentation workflows to support workflow-guided single-source documentation.

**Conclusions:**

Clinical documentation activities within a Comprehensive Cancer Center can likely be realized in a set of three documentation workflows with conditional branching in a modern workflow supporting clinical information system.

## Background

Comprehensive Cancer Center documentation has constituted a major effort in recent years to improve insight into the disease itself and its progression in different patients [1–7]. Systematic documentation as a result of such efforts has resulted in national cancer registries in many countries [6, 7]. In addition, national cancer plans have been established [8, 9] to collect clinical and epidemiological cancer data in databases such as SEER (Surveillance, Epidemiology, and End Results Program) of the National Cancer Institute (NCI). Efforts have been made to standardize datasets and documentation within those registries to support data exchange and nationwide databases [6, 7].

In previous work several options for clinical data reuse with requirements of cancer registry work and quality assurance have been described [10]. We devised a method to split clinical data into documentation categories and packages which are completed in one treatment encounter by one person. We aligned this data with the requirements for cancer registries and quality assurance. A superset of 286 common data elements could be identified [11] which cover clinical documentation, the baseline documentation of German clinical cancer registries (ADT), the requirements for German epidemiological cancer registries (GEKID) and the Bavarian cancer registry law (BCRL). Several documentation scenarios have successfully been implemented in our workflow engine, for example at the Department of Urology [11].

But we noticed significantly different clinical documentation requirements between different departments. This forced us, for example, to modify our schema for melanoma cases where most of the treatment and follow up is carried out on outpatient basis whereas prostate cancer is mainly treated in hospital for many process steps.

Erlangen University Hospital (EUH), a 1361 bed maximum-care facility, has been a certified oncology center within the Deutsche Krebsgesellschaft since 2011. The Comprehensive Cancer Center Erlangen-EMN (CCC-EMN) (located at EUH, with cooperating partner hospitals in Bamberg and Bayreuth) was founded in 2008 as one of 11 German Oncology Excellence Centers and receives funding from Deutsche Krebshilfe e.V. One important CCC-EMN research goal is to establish a single-source-documentation with immediate data capture at the clinical encounter and data reuse for quality assurance, research and cancer registry purposes [11].

EUH runs a hospital-wide commercial Clinical Information System (CIS) including a large variety of clinical assessment forms and computerized physician order entry (CPOE) functionalities, resulting in a comprehensive Electronic Medical Record (EMR). The CIS supports flexible implementation of new documentation forms and comprises a workflow engine to govern clinical activities using digital work lists for doctors and nurses [12–15]. In previous activities we established hospital-wide tumor board documentation and structured treatment planning for all cancer entities [11] besides a so called cancer diary for a fast assessment of long standing cancer cases [4, 11]. A complete comprehensive cancer documentation workflow to support certification and quality control has been established in urology for prostate, bladder and kidney carcinoma [11, 16].

The Erlangen clinical cancer registry associated with EUH operates the Gießen Tumour Documentation System (GTDS) [17, 18] cancer registry system with some interfaces to the clinical documentation at EUH and other regional hospitals.

We discovered that it was not possible to adapt the mentioned implemented electronic documentation forms and the workflow for prostate cancer at our urology department to other cancer entities such as cervical cancer, lung cancer or leukemia. In this situation we recognized that it was essential to know which process steps in diagnostics and therapy of all cancer entities were common or similar or indeed not. Our project was initiated in response to the fact that no global approach could be found in literature or databases. The goals of our study were first to make it possible to use all medical documentation information in accordance with single-source principles for cancer registries and quality assurance without any additional secondary documentation and second to implement electronic documentation forms in our health-information system for a large number of tumour entities.

This situation prompted the following questions:

- Is it really possible to devise one single and unified digital documentation structure for the clinical documentation of all cancer entities within a Comprehensive Cancer Center?
- Is it possible to define a single and all-embracing clinical documentation workflow from first diagnostics to (repeated) therapy and follow-up?
- Alternatively, what is needed to adapt documentation scenarios and documentation workflow for the different cancer entities?

## Methods

Medical specialists and expert groups focused, for their part, on single cancer entities and devised guidelines and clinical pathways for optimal diagnostics and treatment [19–26]. Patient centred clinical documentation is mandatory in most countries in paper based and electronic medical records (EMR) [27, 28]. Guidelines and pathways interact with clinical documentation or may even form the foundation for guideline-based clinical documentation.

Interoperable structures such as the Cancer Biomedical Informatics Grid (caBIG™) [5, 29, 30] have been proposed to combine both types of documentation and to promote a research centred interoperable biomedical informatics infrastructure for management, analysis and dissemination of cancer information. The topic clinical care documentation and its data reuse, however, received limited attention, within the caBIG initiative [5, 29, 30]. Thus, there is still a considerable gap between clinical patient documentation and supplementary data collection for registries, quality assurance and research. This often results in multiple and overlapping documentation activities on various media [1, 31, 32].

Therefore we performed a systematic analysis and final classification of the clinical pathways and documentation requirements including repeated in- and outpatient episodes and different tumour stages at the EUH for 13 representative tumor entities, namely prostate, kidney, bladder, colon, rectum, breast, cervical, lung (small-cell and non-small cell lung cancer), thyroid cancer, melanoma, plasmocytoma, acute myeloid leukemia and lymphoma.

**Fig. 1:**
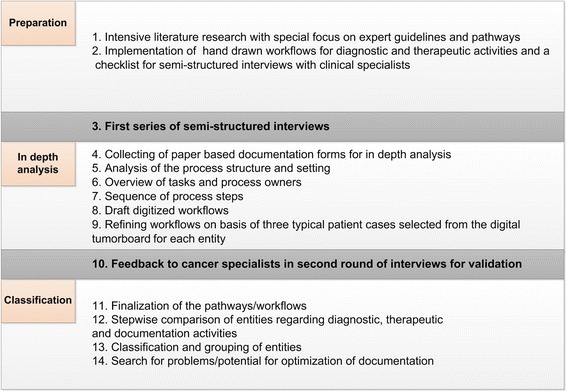
Methodology for the classification and grouping of oncology activities

For this analysis we combined parts of an established process analysis method by Pomberger and Gerken [33–35] with semi-structured interviews, feedback loops and classification methods in a new methodological approach with 14 steps (Fig. 1).

According to local legislation (EUH clinical ethics committee, http://www.ethikkomitee.med.uni-erlangen.de) ethics approval was not needed for our study because none of the following elements was performed:

- interviews with patients or children
- experimental tests
- animal experiments
- clinical trials in humans
- work with animal or human tissues.

Pomberger and Gerken [33, 34] described a method which is useful for analyses designed to implement computer systems in healthcare environments. We selected the following steps, and adapted them for our own methodological purposes:

- structural analysis of diagnostics, therapy and documentation activities connected with them
- analysis of documentation methods: paper or computer systems or even both mixed up
- analysis of process owners
- sequence analysis of the chronological order of process steps and
- analysis of weak points to fix and gain a unified documentation process with computer systems

As a result we combined several popular methods, namely process analysis and workflow modeling techniques with a new focus on a great number of tumour entities instead of single cancer entities described in previous work.

The 14 steps of the methodology comprised:

A comprehensive literature search *(1)* with a special focus on expert guidelines and existing pathways for the selected cancer entities in:

- PubMed
- ScienceDirect
- Medpilot
- Thieme eJournals
- gopubmed.com
- SpringerLink
- UpToDate Inc.
- guidelines of AWMF in Germany (Arbeitsgemeinschaft der Wissenschaftlichen Medizinischen Fachgesellschaften)
- medical expert literature in German language in the field of oncology (Das Rote Buch, Kompendium Internistische Onkologie, Therapiekonzepte Onkologie, Pschyrembel Therapie, Taschenbuch Onkologie 2010/2011).

Data from this step was used to implement a first set of hand-drawn workflow sketches on paper for presumed diagnostic and treatment activities along the clinical pathway of each cancer entity. A checklist was developed for the semi-structured interviews with clinical cancer specialists at the EUH from all departments involved in cancer treatment. They were asked to answer the following main questions *(2)*:

- “Are the workflow graphics complete or are important process steps missing?”
- “When does each step occur?”
- “How long does each step take?”
- “Who is responsible for each part of the process?”
- “How is the process step documented: on paper or in computer systems?” *(2)*.

In step *(3)* we conducted a first series of semi-structured interviews with those cancer specialists participating in the CCC-EMN activities of the respective departments. All interview partners agreed to participate in the study. No human material was used. No individual patient data was integrated. Each interview took between one and two hours for one tumour entity. Therefore at the department of Urology one interview took between three and six hours since three entities are part of it. As we focused on medical decisions in diagnostics and therapy we only interviewed experienced doctors and did not talk to nurses. But we talked to experts for all of our electronic information systems to gather all required content. For each entity two interview series were performed. The experts interviewed were the same in both rounds. Globally 26 interviews were performed.

Within these interviews the typical paper based documentation forms were collected for later analysis: at our departments still 82 paper forms additional to the computer-based documentation systems *(4).*

Especially paper forms for medical history and clinical examination processes as well as chemo- and radiotherapy were collected and taken into account.

The process structure and setting were analyzed in more detail by answering the following questions *(5)*:

- “Where does each process step take place?”
- “At our hospital or outside or is even both possible?”

An overview of tasks and process owners was compiled in form of tables for each department and tumour entity *(6)*. After that a sequence of process steps was derived in a typical chronological order *(7)*. This resulted in a set of draft digitized workflows *(8)* which were refined based on the data from three typical patient cases selected from the digital tumour board for each entity *(9)*.

The workflows were designed within the software package Microsoft Visio Professional 2010. It was selected after several software tests with the workflow modeling program solutions Visio, ARIS, Aris Express, ADONIS and Visual Paradigm for UML. We defined one common design with special icons to be used for all of the 73 different workflow graphics. Furthermore, a set of standardized symbols was drafted. This illustrated all similar activities of the workflows, for example ultrasound or x-ray.

The digitized pathways and results of this stage were fed back to the medical cancer specialists in a second round of interviews *(10)* for validation to answer the question:

- “Are the workflows complete or are important process steps missing?”

The specialists were asked to answer the same questions mentioned above *(2)*.

The following steps comprised finalization of the pathways according to the results of the feedback round *(11)*, stepwise comparison of entities regarding diagnostic, therapeutic and documentation activities according to the following question:

- “Which process steps are similar between the cancer entities and can consequently be implemented in electronic documentation workflow forms without many modifications or even without any modifications?”

The results section focuses on the classification process *(12)*, classification and grouping of the entities following the goal of the step before *(13)* and search for problems/potential for optimization of documentation for all departments *(14)*. The leading questions were:

- “What can be enhanced to help us reach our goal of a completely digital documentation workflow?”
- “How can all medical professions be included in this approach to improve their daily work routine?”

## Results

The preparation of the interviews and the interviews with the clinicians and other staff of the departments themselves were performed successfully. The results section focuses on the classification and grouping process of the tumour entities. These parts are the most important ones to answer the prompted research questions from the beginning.

Due to the extent and volume of the results (724 pages) and the limited length of our paper we will describe only the results of our classification process and what lessons can be learned from it for future workflow implementations at other environments.

The workflow analysis for 13 cancer entities (prostate, kidney, bladder, colon, rectum, breast, cervical, lung (small-cell and non-small cell lung cancer), thyroid cancer, melanoma, plasmocytoma, acute myeloid leukemia and lymphoma) resulted in a total of 73 workflows for the 13 entities. It considered 82 existing paper based cancer documentation forms besides computer systems in a 724 page document [16, 35].

The typical process documentation for one cancer entity comprised between 10 and 16 pages with separate pathways and workflow diagrams for diagnostics, treatment and follow-up processes. Diagnostic pathways were for example specified down to atomic activities such as history and clinical examination, laboratory tests, x-ray-imaging, ultrasound imaging, spirometry, biopsies etc.

We distinguished mandatory and optional activities, depending on the cancer stage. For melanoma patients, for example, a PET-CT (Positron Emission Tomography, computer tomography) is only required in cases with a cancer depth of more than 4 millimetres.

As a result of steps 12 and 13 described in the methods section, the 13 entities were grouped to cancer types in tabulated format, separately for diagnostic (see Table 1 for a condensed version) and therapeutic activities. At this stage, the entities colon and rectum carcinoma were grouped together in one common cancer type with many similarities, and plasmocytoma plus acute myeloid leukemia were grouped in a non-solid cancer type, mainly treated with chemotherapy and stem cell transplant. Within this tabular presentation further classification was done and this yielded common categories (marked boxes in Table 1). These categories subsume similar diagnostic workflows for the entities prostate, kidney and bladder carcinoma respectively breast and cervical carcinoma (Table 1).

**Table 1:**
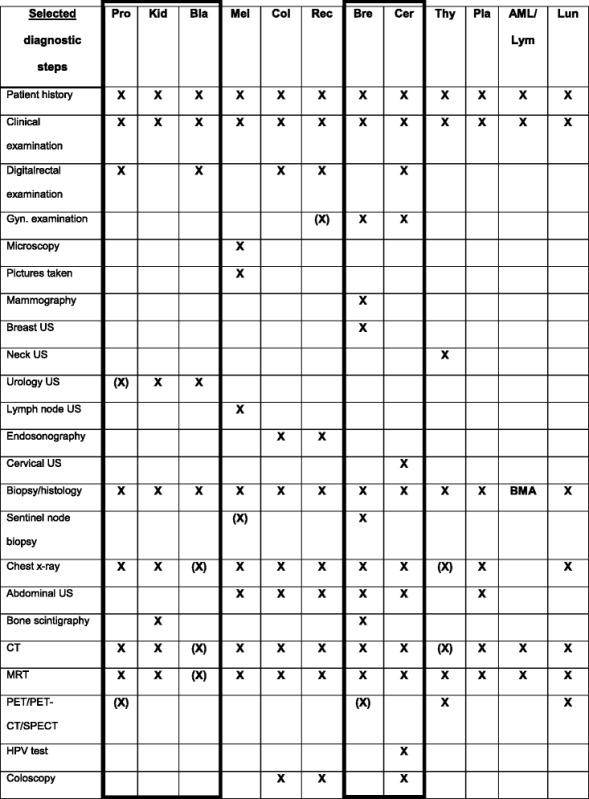
Tumour entities: comparison concerning diagnostic procedures of 13 entities at the CCC Erlangen-EMN

X: obligate steps; (X): ‘facultative’ steps; *BMA* bone marrow aspiration, *Pro* Prostate, *Kid* Kidney, *Bla* Bladder, *Mel* Melanoma, *Col* Colon, *Rec* Rectum, *Bre* Breast, *Cer* Cervical cancer, *Thy* Thyroid, *Pla* Plasmocytoma, *AML* Acute myeloid leukaemia, *Lym* Lymphoma: not analysed separately, *Lun* Lung

Categories with similar process steps and activities obtained at this stage support the assumption that documentation activities can be implemented in common digital forms with minor adaptions to the respective cancer entities.

Table 2 summarizes the categorization results obtained for the diagnostic and therapeutic workflows. Alignment was possible among 5 entity groups with similar therapeutic and diagnostic activity workflows. Thyroid cancer cases and lung cancer cases stand for themselves.

**Table 2:** Deriving groups of combined entities with similar diagnostic or therapeutic activities

Summarized workflow categorization results after grouping to cancer types	
Diagnostic classes	Therapy classes
• Prostate, kidney and bladder cancer	• Prostate, kidney and bladder cancer
• Breast cancer, cervical cancer	• Breast cancer, cervical cancer
• Melanoma	• Melanoma
• Cancer of colon and rectum	• Cancer of colon and rectum
• Lung, thyroid cancer	• Lung cancer: small cell and non-small cell lung cancer
• no corresponding diagnostic type	• Thyroid cancer
• Acute myeloid leukemia, plasmocytoma	• Acute myeloid leukemia, plasmocytoma

**Fig. 2:**
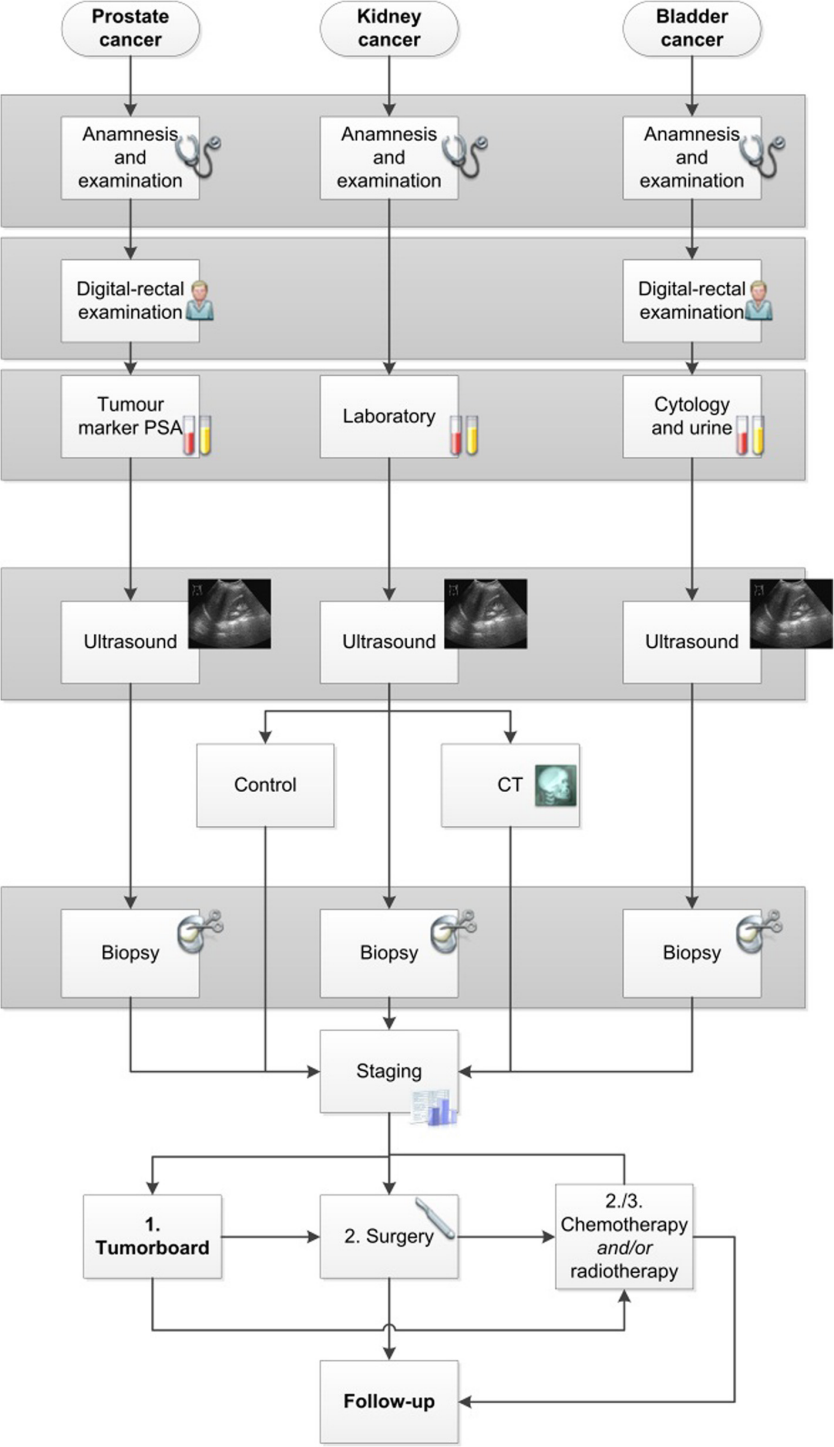
Grouping of diagnosis and therapy for prostate, kidney and bladder

In a subsequent step we assembled condensed, top level, grouped pathway diagrams (Fig. 2). These grouped diagrams preserve the essential differences between the entities, for example, no digital rectal examination in kidney cancer cases, and computer tomography (CT) staging for some kidney cases, due to the fact that early kidney cancer stages can be cured by surgery, whereas advanced stages require combinations of surgery, radiation and chemotherapy, adapted to the individual patient. The potential combinations had been analysed in advance according to the UICC (Union for International Cancer Control) - and AJCC (American Joint Committee on Cancer) –systems. Follow-up intervals and procedures were collected for all entities and arranged in special tables.

With those grouped pathway diagrams and the process entities included we can derive the basis for common clinical cancer documentation on a level of sufficient detail for subsequent use in quality assessment, certification, cancer registries, studies or other research.

A grouped pathway would correspond to a common set of CIS documentation forms and a common documentation pathway with minor branching to accommodate such specific requirements as the mentioned rectal examination or the CT staging in some of the cases. Branching within the grouped workflow is also required for different tumour classification schemes or different cancer follow-up timetables in the respective entities. It was not possible to gain only one class for all entities because we realized that the prostate cancer workflow, for example, was not transferable to melanoma or breast cancer since the components of the process were too different to get only one group for all of them. For melanoma you have other tumour markers and incident light microscopy. For prostate you have a digital-rectal examination for example, but not for melanoma.

First of all we identified two main groups after finishing the classification process:

- solid and
- non-solid-entities (plasmocytoma and acute myeloid leukemia)

In a next step we realized that in early stages surgery may be the only therapeutic component of a process (see melanoma or kidney cancer patients) and if there were metastases we would have combinations of surgery, radio- and chemotherapy (see breast cancer or cervical cancer for example).

**Fig. 3:**
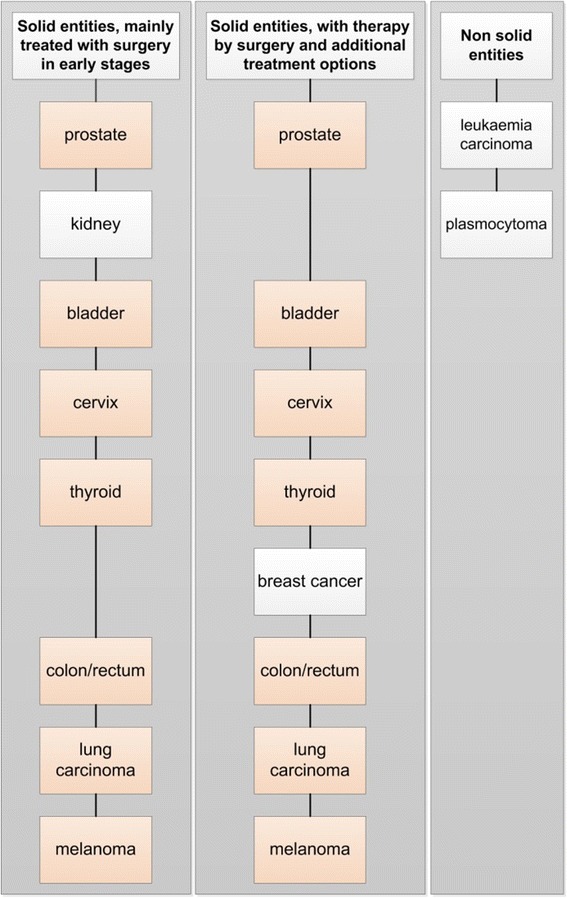
Grouping tumour entities to three classes

Grouped pathway diagrams were possible for three major classes of tumour entities (Fig. 3), namely

- *Solid entities with surgical therapy* comprising prostate, kidney, bladder, cervical, colon and lung carcinoma. Typically, therapy starts with inpatient surgery and continues on an ambulatory basis.
- *Solid entities with surgical therapy and additional therapeutic activities*, e. g. thyroid carcinoma surgery combined with inpatient radio-chemotherapy. Or melanoma, often treated with ambulatory surgery and outpatient follow-up.
- *Non solid entities* such as plasmocytoma and leukemia typically treated initially with inpatient chemotherapy.

As a next step following the classification process we focus on the development of corresponding unified documentation workflows.

A total of 73 workflows for the 13 entities based on 82 paper documentation forms were compiled in a 724 page document comprising 130 figures, 94 tables and 23 tumour classifications and 12 follow-up tables.

The identified three classes should be recognized when implementing clinical documentation modules because they show some important differences between the entities. Furthermore, the order of inpatient and outpatient episodes is too different that a single clinical documentation module which would strive to cover all three would have to support inpatient and (cross-institutional) outpatient documentation activities alike. For example, remember melanoma cancer with treatment mainly on ambulatory basis, compared to leukemia mainly treated in hospital. The first and the third class are discrepant with regard to the fact that for the surgical entities close integration with the surgical planning and documentation systems and bidirectional exchange of highly structured data is required.

Outpatient data connected with the processes and patient cases is collected by a web-based application from external or by our own hospital doctors, for example, during the follow-up talks at our department (manual data input into our CIS).

Construction of single source clinical cancer documentation thus started as follows: For each of the three classes we defined a common and intrinsic documentation pathway for diagnostics, therapy and follow-up which caters for the specific entity and tumour stage related diagnostics and treatment options for each affected entity. These options were mirrored in conditioned branches within forms and the workflow engine of the CIS as a guide through the great amount of documentation forms. At any time, the generalized pathway may be complemented with additional stage and entity specific sub workflows according to patient-specific decisions by the interdisciplinary tumour board sessions.

To substantiate this approach we analyzed the current data processing environment to advance the unification of cancer documentation, e.g. for solid cancer entities. An overview of diagnostics and staging workflow is given in Table 3.

**Table 3:** Process steps in electronic documentation suitable for a unified documentation in diagnostics

Process steps in documentation suitable for a unified documentation in diagnostics	Urology	Surgery	Gynecology	Pulmonology/gastroenterology/thoracic surgery	Radiotherapy	Hematology/oncology
History	S/F	P	P	P/S	P	P
Examination	S/F	P	P	P/S	P	P
Biopsy	M/S/P	M	P	M/S	-	S
x-raying	W/F	W	W	W	W/P	W
Ultrasound	S/P/F	Plaza	V	V	-	-
CT	W/F	W	W	W	P	W
MRT	W	W	W	W	P	W
FDG-PET/SPECT	W	W	W	W	-	W
Laboratory examination	L	L	L	L	L	L

*S* clinical information system, *P* paper based documentation

*L* laboratory information system, *W* web-based radiology application

*V* application for storing ultrasound pictures

*M* program for surgery reports

*F* own clinical information system of the Waldkrankenhaus St.Marien Erlangen

“-” = not used, *Plaza* part of our radiology information system

Five clinical departments are primarily responsible for the treatment of the solid entities with surgical therapy prostate, kidney, bladder, cervical, colon and lung carcinoma: The departments of urology, general surgery, gynecology, pulmonology and thoracic surgery. Patients with advanced disease however undergo primary treatment in the departments of radiotherapy and oncology.

Table 3 demonstrates that, at the time of the study, some IT applications within the CIS were already unified: E.g. the browser-based application for radiology CPOE (W) is available in all departments. Biopsy reports are mostly written in the same surgical department application MCC.NET™. At the time however, radiotherapy documentation was largely paper based (apart from radiotherapy planning software applications and Lantis™), indicating that there was room for improvement.

We implemented electronic documentation workflows and documentation forms for all steps of the diagnostic and therapeutic processes in our workflow engine of the CIS. After this important step we evaluated the effects on our hospital organization and the daily documentation workload of the doctors, compared to the mixed-up documentation structures before.

To sum up the main impacts of our workflow implementation we noticed a reduced documentation time, higher quality in the documented fields and its contents as well as a greater database for reuse of our clinical routine data in our cancer registry after export from the CIS.

In the fields of medical history and clinical examination data quality could be improved most by replacing all different sorts of paper forms through one single branched electronic version for all departments. The same effect applied to radio- and chemotherapy planning as well as the treatment cycles.

The documentation workload of our doctors could be reduced by nearly 25 per cent. Moreover, the satisfaction of our doctors regarding clinical documentation itself could be increased by our approach. But finally even the patients profit from the greater database of structured information at the EUH since we can easily reuse existing data for research or certification without additional efforts to provide the best treatment options.

## Discussion

The prompted research questions from the beginning could be answered as follows:

- Is it really possible to devise one single and unified digital documentation structure for the clinical documentation of all cancer entities within a Comprehensive Cancer Center?

It is possible to cover all cancer entities with one electronic documentation workflow engine system solution. But you need three classes of entities to deal with all exceptions of the diagnostic and therapeutic processes. One class alone would not be able to solve this approach.

- Is it possible to define a single and all-embracing clinical documentation workflow from first diagnostics to (repeated) therapy and follow-up?

We stated examples from our processes to show that this is possible for medical history, clinical examination, laboratory, imaging techniques and biopsy as well as surgery and radio- or chemotherapy. As a condition, however, you need the same electronic documentation environment/system for each step at all departments of the hospital and adjustable electronic documentation forms to cover differences between the entities by branching. In addition, paper forms have to be replaced by electronic equivalents completely.

- Alternatively, what is needed to adapt documentation and documentation workflow for the different cancer entities?

In the complete report of our project (724 pages) [35] we described the details to adapt documentation methods for the unification of all similar process steps in diagnostic and therapy.

The first steps for a unified application are the unification of data source systems and especially elimination of non-digital sources. In our case, the department of radiotherapy underwent a complete redesign of information flow and clinical documentation, which has resulted in digital documentation of history and examination within the system plus digitizing and PACS (picture archiving and communication system) connection for the planning CT to adapt for a future unified documentation. In our case study the urology department runs two clinical information systems because it has two different locations in the city of Erlangen.

There have, in the past, been many efforts to introduce, support and standardize clinical cancer documentation using single-source approaches or other thoughts [1, 10, 11, 28, 30, 36–38]. Existing standards such as HL7 and other specifications for data exchange between medical service providers alone are not able to fill the gap und to achieve a flexible and adjustable workflow-guided tumour documentation of all medical steps at a CCC. Current German guidelines or recommendations in literature [39–42] are mostly text based and static and thus, unfortunately, unable to particularly support clinical pathways with documentation workflows [43, 44].

IT supported clinical pathways [28, 37, 45–51] are still scarce and only few implementations have made it into routine clinical use. In addition, many approaches did not result in wide applicability and comprehensive data reuse for many purposes, but concentrated on specific needs, for example bioinformatics purposes and molecular research in the case of the Cancer Biomedical Informatics Grid™ (caBIG™) [5, 29, 30].

Registry based documentation systems such as GTDS or other center focused cancer documentation systems rarely support guidelines or workflows and demand extra documentation activities outside the regular hospital CIS for cancer cases, which limits their usability [16, 31, 52, 53].

With regard to the methods we combined several popular methods, namely process analysis [33, 34] and workflow modeling techniques with a focus on a great number of tumour entities instead of single cancer entities described in previous work.

Within this paper we have devised an approach which starts from the single-source paradigm and data reuse for following activities. Besides, it considers the typical CIS as the primary data source which shall be adapted for these purposes. We combined a clinical pathway analysis and the subsequent categorization and classification steps to obtain grouped pathway diagrams for cancer diagnostics and treatment.

Final we came to the conclusion that

- one single static clinical documentation module will not be sufficient to cover the documentation requirements for all cancer entities by applying only one class alone without branching entities to groups.
- It is however possible to construct a complete clinical documentation workflow in a three tier approach dependent on the tumour type.
- Within one tier, branching options of the CIS forms and workflow engine can potentially support the adaption to different entities and cancer stages.

Thus we hope to be able to guide clinicians with restricted effort through the jungle of treatment and documentation and to promote the right documentation activities at the right time. Our workflow model will manage the appearance of electronic documentation forms in our CIS for all process steps of all patients at the EUH from initial diagnostics, treatment to follow-up. The developed workflow model takes care of changing and optimized or even new therapeutic standards in radio- or chemotherapy. We provide flexible structures for chemotherapy and radiotherapy schemes that adapt to the patient’s individual case/stage and focus on other conditions during treatment process. Therefore, it is easy to integrate new therapeutic knowledge within our workflow model.

Our goal is a structured clinical documentation network for cancer cases, covering history/diagnostics, therapy and follow-up activities for the different CCC-EMN cancer entities. The CIS workflow would be activated through either a patient diagnosis or the tumour board decision. Then the user will be guided to the documentation steps at the specific time, with reminders for “forgotten” activities. Many steps in the analysed pathways are conditional, in the sense that previous diagnostic or therapeutic activities have to be finished first. Facultative procedures would be offered as an option depending on individual cancer stage and patient’s characteristics. Some steps, however, do not have a fixed order which must be respected in the workflow engine. It does not matter whether an ultrasonic examination is done before or after an x-ray e. g., if both are needed for proceeding to the next workflow steps, for example for colon/rectum cancer.

The model of grouped workflows can potentially be transferred to other environments, especially other Comprehensive Cancer Centers, because the pathways are originally based on expert guidelines and literature [16]. Furthermore, they have been quality checked for completeness and ambiguity during repeated interviews with experienced clinical specialists.

## Conclusions

The findings of this investigation may be a guideline for other Cancer Centers on the way to workflow- based single source cancer documentation aligned with current diagnostics and treatment pathways. We may state that grouped pathways for three categories of cancer diseases in combination with a sufficiently flexible CIS should enable the realization of clinical cancer documentation for all entities of a Cancer Center. It will remain an interesting task for the future, however, to see how disparate CIS in the different ambulatory and inpatient institutions could be linked within such pathways. With regard to methodological aspects this study describes a new combined 14 step method designed to derive, categorize and classify common, grouped clinical pathways for cancer patients. The study confirms that such grouped clinical pathways may be translated into documentation modules with conditional branching. They may be realized within the forms and workflow engine of a modern Clinical Information System (CIS).

### Ethics approval and consent to participate

No human material was used. No individual patient data was integrated.

Not applicable.

According to local legislation (EUH clinical ethics committee, http://www.ethikkomitee.med.uni-erlangen.de) ethics approval was not needed for our study because none of the following elements was performed:

- interviews with patients or children
- experimental tests
- animal experiments
- clinical trials in humans
- work with animal or human tissues.

## Abbreviations

NCI: National Cancer Institute
CIS: Clinical information system
CCC-EMN: Comprehensive Cancer Center Erlangen-European Metropolregion Nuremberg
CT: Computer tomography
PET: Positron Emission Tomography
AJCC: American Joint Committee on Cancer
UICC: Union for International Cancer Control
GTDS: Gießen Tumour Documentation System
ADT: Baseline documentation of German clinical cancer registries
GEKID: German epidemiological cancer registries
BCRL: Bavarian cancer registry law
EUH: Erlangen University-Hospital
EMR: Electronic medical record
SEER: Surveillance, Epidemiology, and End Results Program
BMA: Bone marrow aspiration
Pro: Prostate
Kid: Kidney
Bla: Bladder
Mel: Melanoma
Col: Colon
Rec: Rectum
Bre: Breast cancer
Cer: Cervical cancer
Thy: Thyroid
Pla: Plasmocytoma
AML: Acute myeloid leukaemia
Lym: Lymphoma
Lun: Lung
PACS: Picture archiving and communication system
caBIG: Cancer Biomedical Informatics Grid

## References

[1] Singprasong R, Eldabi T. An integrated methodology for process improvement and delivery system visualization at a multidisciplinary cancer center. J Healthc Qual. 2011. doi:10.1111/j.1945-1474.2011.00174.x. 10.1111/j.1945-1474.2011.00174.x 22092497

[2] Organization WH. WHO | Global cancer rates could increase by 50 % to 15 million by 2020. 2003. http://www.who.int/mediacentre/news/releases/2003/pr27/en/. Accessed 08.04.2011.

[3] Krumm R, Semjonow A, Tio J, Duhme H, Bürkle T, Haier J (2014). The need for harmonized structured documentation and chances of secondary use - results of a systematic analysis with automated form comparison for prostate and breast cancer. J Biomed Inform.

[4] Ries M, Golcher H, Prokosch H-U, Beckmann MW, Bürkle T (2012). An EMR based cancer diary - utilisation and initial usability evaluation of a new cancer data visualization tool. Stud Health Technol Inform.

[5] Califano A, Chinnaiyan A, Duyk GM, Gambhir SS, Hubbard T, Lipman DJ (2011). An assessment of the impact of the NCI cancer biomedical informatics grid (caBIG®): Report of the Board of Scientific Advisors Ad Hoc Working Group.

[6] Bilimoria KY, Stewart AK, Winchester DP, Ko CY (2008). The National Cancer Data Base: a powerful initiative to improve cancer care in the United States. Ann Surg Oncol.

[7] Government A. A national cancer data strategy for Australia. Barton. https://canceraustralia.gov.au/sites/default/files/publications/ncds_final_web1_504af02093a68.pdf.

[8] Evrard S (2010). Enhancing patient safety and quality of surgical cancer care: the French National Cancer Plans. Eur J Surg Oncol.

[9] Zylka-Menhorn V (2008). Nationaler Krebsplan: Startschuss für einen mehrjährigen Prozess. DÄ.

[10] Prokosch H-U, Ries M, Beyer A, Schwenk M, Seggewies C, Köpcke F (2011). IT infrastructure components to support clinical care and translational research projects in a comprehensive cancer center. Stud Health Technol Inform.

[11] Ries M, Prokosch H-U, Beckmann MW, Bürkle T (2013). Single-source tumor documentation - reusing oncology data for different purposes. Onkologie.

[12] Haux R (2006). Health information systems - past, present, future. Int J Med Inform.

[13] Haux R (2006). Individualization, globalization and health - about sustainable information technologies and the aim of medical informatics☆. Int J Med Inform.

[14] Haux R, Häber A (1998). Management von Informationssystemen: Analyse, Bewertung, Auswahl, Bereitstellung und Einführung von Informationssystemkomponenten am Beispiel von Krankenhausinformationssystemen. Leitfäden der Informatik.

[15] Haux R, Seggewies C, Baldauf-Sobez W, Kullmann P, Reichert H, Luedecke L (2003). Soarian - workflow management applied for health care. Methods Inf Med.

[16] Bürkle T, Martin M, Schütz A, Starke K, Wagner S, Ries M (2013). Workflows in cancer treatment and their influence upon clinical documentation. Stud Health Technol Inform.

[17] Altmann U. Gießener Tumordokumentationssystem (GTDS). 2011. http://www.med.uni-giessen.de/akkk/gtds/. Accessed 08.04.2011.

[18] Altmann U, Katz FR, Dudeck J (2006). A reference model for clinical tumour documentation. Stud Health Technol Inform.

[19] Garde S, Kutscha U, Merzweiler A, Weber R, Knaup P (2004). Integration von Therapieplanung und standardisierter Dokumentation - Ergebnisse aus der Entwicklung und Einführung eines rechnerbasierten Anwendungssystems der Pädiatrischen Onkologie: Forschungsbericht Nr. 01/2004 der Abteilung Medizinische Informatik.

[20] Carvalho ECA, Batilana AP, Claudino W, Reis LFL, Schmerling RA, Shah J (2012). Workflow in clinical trial sites & its association with near miss events for data quality: ethnographic, workflow & systems simulation. PLoS One.

[21] Carvalho ECA, Jayanti MK, Batilana AP, Kozan AMO, Rodrigues MJ, Shah J (2010). Standardizing clinical trials workflow representation in UML for International Site Comparison. PLoS One.

[22] Lester WT, Ashburner JM, Grant RW, Chueh HC, Barry MJ, Atlas SJ (2009). Mammography fasttrack: an intervention to facilitate reminders for breast cancer screening across a heterogeneous multi-clinic primary care network. J Am Med Inform Assoc.

[23] Bittner R, Burghardt J, Gross E, Grundmann RT, Hermanek P, Isbert C (2007). Bericht über den Workshop “Workflow Rektumkarzinom II” am 10./11.11.2006 in Burghausen. Zentralblatt für. Chirurgie.

[24] Buhr HJ, Dommisch K, Fleischer G-M, Gastinger I, Grundmann RT, Hermanek P (2006). Klinischer Ablaufpfad (Workflow) zu Diagnostik, Therapie und Nachsorge des Rektumkarzinoms. Zentralbl Chir.

[25] Grundmann R, Hölscher A, Bembenek A, Bollschweiler E, Drognitz O, Feuerbach S (2009). Diagnostik und therapie des Magenkarzinoms – workflow. Zentralbl Chir.

[26] Grundmann RT, Hermanek P, Merkel S (2008). Diagnostik und Therapie von Lebermetastasen kolorektaler Karzinome - workflow. Zentralbl Chir.

[27] Bowens FM, Frye PA, Jones WA (2010). Health information technology: integration of clinical workflow into meaningful use of electronic health records. Perspect Health Inf Manag.

[28] Séroussi B, Bouaud J, Antoine É-C (2001). OncoDoc: a successful experiment of computer-supported guideline development and implementation in the treatment of breast cancer. Artif Intell Med.

[29] Eschenbach AC, Buetow K (2006). Cancer informatics vision: caBIG. Cancer Inform.

[30] Fenstermacher D, Street C, McSherry T, Nayak V, Overby C, Feldman M. The Cancer Biomedical Informatics Grid (caBIG™): Proceedings of the 2005 IEEE Engineering in Medicine and Biology 27th Annual Conference Shanghai, China, September 1–4, 2005. 2005 27th annual international conference of the IEEE Engineering in Medicine and Biology Society. 2005;1:743–6. 10.1109/IEMBS.2005.1616521 17282290

[31] Davidson SJ, Zwemer FL, Nathanson LA, Sable KN, Khan ANGA (2004). Where’s the Beef? The promise and the reality of clinical documentation. Acad Emerg Med.

[32] Brucker SY, Bamberg M, Jonat W, Beckmann MW, Kämmerle A, Kreienberg R (2009). Certification of breast centres in Germany: proof of concept for a prototypical example of quality assurance in multidisciplinary cancer care. BMC Cancer.

[33] Pomberger G (1987). Softwaretechnik und Modula-2. 2 ed. Hanser-Studien-Bücher.

[34] Gerken W (1988). Systemanalyse: Entwurf und Auswahl von DV-Anwendersystemen.

[35] Wagner S (2015). Analyse, Modellierung und Optimierung der Dokumentations-, Diagnostik- und Therapieprozesse für verschiedene Tumorerkrankungen am Universitätsklinikum Erlangen.

[36] Ammenwerth E, Spötl H-P (2009). The time needed for clinical documentation versus direct patient care: a work-sampling analysis of physicians’ activities. Methods Inf Med.

[37] Tafazzoli AG, Altmann U, Bürkle T, Hölzer S, Dudeck J (2002). Integrated decision support in a hospital cancer registry. Artif Intell Med.

[38] Dugas M, Breil B, Thiemann V, Lechtenbörger J, Vossen G (2009). Single source information systems to connect patient care and clinical research. Stud Health Technol Inform.

[39] Berger DP, Engelhardt R, Mertelsmann R, Engelhardt M (2010). Das Rote Buch: Hämatologie und Internistische Onkologie.

[40] Preiß J, Bettag M (2010). Taschenbuch Onkologie: Interdisziplinäre Empfehlungen zur Therapie 2010/2011.

[41] Schmoll H-J, Höffken K, Possinger K (2006). Kompendium Internistische Onkologie: Standards in Diagnostik und Therapie.

[42] Seeber S, Schütte J (2003). Therapiekonzepte Onkologie.

[43] De Clercq P, Blom JA, Korsten HHM, Hasman A (2004). Approaches for creating computer-interpretable guidelines that facilitate decision support. Artif Intell Med.

[44] De Clercq P, Kaiser K, Hasman A (2008). Computer-interpretable Guideline Formalisms. Stud Health Technol Inform.

[45] Bürkle T, Castellanos I, Tech H, Prokosch H-U (2010). Implementation of a patient data management system - an evaluation study of workflow alterations. Stud Health Technol Inform.

[46] Dickhaus CF, Burghart C, Tempany C, D'Amico A, Haker S, Kikinis R (2004). Workflow modeling and analysis of computer guided prostate brachytherapy under MR imaging control. Stud Health Technol Inform.

[47] Halsted MJ, Froehle CM (2008). Design, implementation, and assessment of a radiology workflow management system. AJR Am J Roentgenol.

[48] Kalinski T, Sel S, Hofmann H, Zwönitzer R, Bernarding J, Roessner A (2008). Digital workflow management for quality assessment in pathology. Pathol Res Pract.

[49] Ye Y, Jiang Z, Diao X, Yang D, Du G (2009). An ontology-based hierarchical semantic modeling approach to clinical pathway workflows. Comput Biol Med.

[50] Bürkle T, Baur T, Höss N (2006). Clinical pathways development and computer support in the EPR: lessons learned. Stud Health Technol Inform.

[51] Bürkle T, Kuch R, Prokosch HU, Dudeck J (1999). Stepwise evaluation of information systems in an university hospital. Methods Inf Med.

[52] Breil B, Semjonow A, Dugas M (2009). HIS-based electronic documentation can significantly reduce the time from biopsy to final report for prostate tumours and supports quality management as well as clinical research. BMC Med Inform Decis Mak.

[53] Sonnenberg FA, Hagerty CG (2006). Computer-Interpretable Clinical Practice Guidelines Where Are We and where Are We Going?. Yearb Med Inform.

